# Promoting Students' Health at University: Key Stakeholders, Cooperation, and Network Development

**DOI:** 10.3389/fpubh.2021.680714

**Published:** 2021-06-30

**Authors:** Philip Bachert, Hagen Wäsche, Felix Albrecht, Claudia Hildebrand, Alexa Maria Kunz, Alexander Woll

**Affiliations:** ^1^Institute of Sports and Sports Science, Karlsruhe Institute of Technology, Karlsruhe, Germany; ^2^Central Scientific Institution for Key Competencies, Karlsruhe Institute of Technology, Karlsruhe, Germany

**Keywords:** organizational network analysis, health-promoting universities, university students' health, cooperation, stakeholder identification, network governance, network development research

## Abstract

**Background:** Cooperation among university units is considered a cornerstone for the promotion of students' health. The underlying mechanisms of health-promoting networks at universities have rarely been examined so far. Shedding light on partnerships is generally limited to the naming of allied actors in a network.

**Objectives and Methods:** In this study, we used network analysis intending to visualize and describe the positions and characteristics of the network actors, and examine organizational relationships to determine the characteristics of the complete network.

**Results:** The network analysis at hand provides in-depth insights into university structures promoting students' health comprising 33 organizational units and hundreds of ties. Both cooperation and communication network show a flat, non-hierarchical structure, which is reflected by its low centralization indices (39–43%) and short average distances (1.43–1.47) with low standard deviations (0.499–0.507), small diameter (3), and the non-existence of subgroups. Density lies between 0.53 and 0.57. According to the respondents, the University Sports Center is considered the most important actor in the context of students' health. Presidium and Institute of Sport and Sports Science play an integral role in terms of network functionality.

**Conclusion:** In the health-promoting network, numerous opportunities for further integration and interaction of actors exist. Indications for transferring results to other universities are discussed. Network analysis enables universities to profoundly analyze their health-promoting structures, which is the basis for sustained network governance and development.

## Introduction

### Problem Statement and Relevance

Despite their young age, university students are a vulnerable group from a health perspective (1–3). Because of the potential multiplier role of university students as future leaders and decision makers, health promotion in higher education institutions is of special importance (4). Because universities are complex organizations, systematically navigating health promotion is necessary for it to be effective and efficient (5).

Therefore, health-promoting universities are being called upon to work according to the *setting approach*, which means that relevant stakeholders from different disciplines and sectors within the campus community should be cooperatively involved in the process of embedding health into all aspects of campus culture and of providing health-promoting activities for students (6, 7). Collective action by a wide range of stakeholders has been seen as a key for effective intervention delivery in health promotion since a single stakeholder can hardly be in control over the complex interplay of determinants of a targeted population's health (8–10).

By cooperating, stakeholders can attain and provide additional resources, share information and knowledge, minimize the duplication of effort, reach additional members of the target audience, earn greater credibility, and tackle the determinants of health in a holistic approach through the provision of integrated services (8, 11–14). However, stakeholders from various disciplines with unique expertise, interests, values, and expectations may not have a history of working together or even view themselves as having related goals, making setting-based health promotion a difficult undertaking (15–17).

### State of Research and Research Gap

Cooperation processes and structural characteristics of various public health networks have been studied in the past, including active living networks (18), healthcare and patient safety networks (19), community academic partnerships for health (20), community care networks (21, 22), substance abuse prevention networks (11, 23), children's health initiative coalitions (24), elderly care networks (25), HIV/AIDS service organizations (26), mental health services (27, 28), woman organizations (29, 30), and cancer support networks (31).

The number of colleges and universities promoting health for students is rapidly increasing (32). The underlying mechanisms of health-promoting networks at universities, however, have rarely been examined so far, and that although multiservice cooperation among the university community is considered a cornerstone for the promotion of health in the university setting (4, 33). In their study on implementation status quo of the health-promoting university concept, Suárez-Reyes et al. (34) have pointed out that “the key principles of health-promoting universities and the framework for action, along with the key components for their implementation, are clearly described, but information on how universities make use of these guidelines to operate in a real context is scarce.” Newton et al. (32) stated in their study on the operationalization of the concept of healthy universities that there is a need for a whole-university approach that pays attention to the complex interactions and interconnections between component parts and highlights how the organization can function effectively as a social system. Reviews have indicated that cooperative practice among units of the university does seem to take place in the context of student health (35, 36), but evidence about communication and cooperation among units promoting health, especially for university students, is almost non-existent, while other aspects of promoting students' health at university are relatively well-studied (35–37). A multi-methodical but not network analytic approach to map out and characterize health-promoting structures was used at the Florida International University (USA) (38). Here, information on localization, resources, and partnerships of health promotion initiatives was collected *via* semi-structured interviews with stakeholders in health-related roles among other things. Shedding light on partnerships, however, is then limited again, as is commonly the case (39, 40), to the naming of allied actors, and does not provide in-depth information about structural characteristics of networks promoting health at university.

### Theoretical Background

The present network analysis falls into the research branch of *organizational network analysis* (41). An organization can be conceptualized as a network in which organizational members or units (consisting of the major representatives of those organizations for example) are nodes interacting with each other, establishing relationships (42). These networks between organizational units are referred to as *intraorganizational networks*, as opposed to *interorganizational networks*, where the focus is on networks between different organizations (43, 44).

Within the research branch of *organizational network analysis*, the present network analysis belongs to the category of *network development research*. Here, so-called *network structure constructs* at all three levels (node, dyadic, and network) are utilized to capture detailed structural features of networks (45). By capturing the structural features of a network, network structure constructs can help to understand the positions and roles of actors and indicate the available opportunities for progress in the network (46).

### Purpose

In this study, we used network analysis with the aim to

- visualize and describe the positions and characteristics of the network actors to identify key-stakeholders;
- examine organizational relationships to determine the characteristics of the complete network; and
- explore the network structures to designate starting points for network development.

The research questions are as follows:

- Which actors are relevant concerning student health?
- How is communication and collaboration between actors structured in the network?
- Which network-related optimization potentials can be identified?

## Methods

### Setting

To address student health issues at the German university at hand, the Institute of Sports and Sports Science and the Central Scientific Institution for Key Competencies launched a participatory health promotion project focused on identifying barriers and opportunities related to integrating evidence-based health promotion programs offered on the university campus in partnership with the Presidium, the Techniker Krankenkasse (German health insurance), Student Support Service, University Sports Center, and student representatives. The university has a long history of health promotion regarding staff members (corporate health management) and partially regarding university students (e.g., health-related courses at the University Sports Center or key qualifications for coping with academic stress). However, a holistic management approach for the promotion of students' health was undertaken at the beginning of this project in 2017. Stakeholders of the project agreed on developing a community-based participatory research approach (47). Through cooperation with the different stakeholders at the university, it was expected that structural change could be implemented more efficiently. Some of these actors provide health promotion or education activities; others were not traditionally associated with health and academic stress themes. This paper reports the findings from a network analysis among actors of the university, which was conducted after the project had been in operation for about 2 1/2 years. The network analysis primarily provides data on the extent to which actors interacted with one another in the network.

### Sampling

To identify all actors that address student health at university, a multifaceted snowball sampling process was initiated (16, 48, 49). First, a pre-defined list was created by the researchers based on the research of project proposals and documents and a screening of the literature. Then, the head managers from the participatory health promotion project for students from the Institute of Sports and Sports Science and the Central Scientific Institution for Key Competencies were asked as key informants to identify the actors with a unique role and others they deemed relevant in the area of health promotion at the university. This resulted in a final sample of 33 actors, who focus on understanding or promoting the health of students at university or who are potentially able to influence student health. The actors were quite diverse. Some of them were actual health providers, others provided health-related information and education, and still others had only indirect involvement with students' health. Fourteen of these organizations were engaged in the project at the time (*via* membership of the steering committee or through engagement in the working group), and the rest was identified as potentially relevant.

### Questionnaire

The questionnaire developed was based on previous work on health- and physical activity-related networks done (16, 49–52). It requested basic information on the estimation of health topics and potency of actors but focused primarily on obtaining information on relationships regarding communication and cooperation among the actors. The questionnaire comprised 18 questions. The quantitative relational constructs measured among the university units were communication and cooperation, operationalized as the frequency of contact and type of cooperation. For each question, a list of the 33 actors was provided. Regarding *communication*, respondents were asked to indicate, how often they are in contact with all of the 33 actors. Communication frequency response options ranged from “never” (0), “less than annually” (1), “annually” (2), “half-yearly” (3), “monthly” (4), “weekly” (5), to “daily” (6). In matters of cooperation, respondents were asked how they would describe their relationship with each of the 33 actors. The cooperation response scale ranged from no cooperation (0); information sharing only (1); informal cooperation (loose cooperation to reach common objectives) (2); formal cooperation (close cooperation in a team to reach common objectives) (3); partnership (close cooperation for longer time period, e.g., in several projects) (4). In order to identify further starting points for network governance and development, respondents were additionally asked about their points of contact regarding their area of work with several health-related topics, perceived importance of these health topics for student health (on a five-point Likert scale from 1 = unimportant to 5 = very important), the relevance of the other actors regarding health topics, and the importance of the other actors regarding student health *per se* (on a five-point Likert scale from 1 = unimportant to 5 = very important). Health-related topics were identified by scanning the research field of health-promoting universities with a focus on students. Apart from that, questions were asked about service duties (e.g., freedom of choice), staffing level, and the employment relationship (*Note: The analysis of these questions is not part of this publication*). The respondents were also given the opportunity to list further relevant actors and health topics, which were not included in the list and which they thought were relevant to students' health. Most questions and answers were administered with accompanying definitions and examples. The questionnaire was prefaced with instructions and data protection information and was piloted with the head of the Corporate Health Management and the deputy managing director of the Central Scientific Institution for Key Competencies.

### Data Collection

Quantitative and qualitative organizational network data were collected during winter semester 2019/2020 by highly structured face-to-face interviews from trained research assistants using an interview guide in an interactive format with actor and health topic lists and response scale cards. The main representative of each of the 33 units (generally the executive director or, in some cases, a staff member who was more knowledgeable about the issue) received a personalized interview request for this purpose, including a cover letter explaining the research study and a privacy statement. Individuals were known from most units; otherwise, contact persons were researched at the homepages of the units. Informed written consent was obtained from all respondents before the start of the interview. The average interview lasted about 60 min. All in all, data collection took 6 months. Approval for this study was granted by the staff council and the data protection office of the university as well as the staff council of the Student Support Service. In the end, 28 out of 33 units completed the survey providing an 85% response rate. Three of the 33 units (Student Groups, Deaneries, and Institutes) represented a collective of various actors and were therefore not interviewed. The General Student Committee and the Student Working Group for Culture and Communication were not available for an interview. In total, 35 persons were interviewed, since the Institute of Sports and Sports Science (three respondents), the Central Scientific Institution for Key Competencies (five respondents), and the Student Support Service (two respondents) in their roles as central stakeholders in the context of student health had more than one respondent.

### Data Analysis

Survey data gathered through the questionnaire were entered to SPSS 25 Statistical Package by study ID for cleaning and initial data exploration on the basis of a codebook. Ten percent of data were randomly double-checked for accuracy—the agreement was 100%, why a higher double-check was refrained from. Data from the two network questions were then exported into Microsoft Excel for the creation of adjacency matrices, indicating which actors reported links of cooperation and communication to other actors. To reconcile divergent response pairs, two techniques were used: reconstruction (when only one actor in the dyad provided a valid response to a question, response given by the other actor in the pair was used) and symmetrizing (minimization was used to resolve rating discordances between two actors in a dyad). When both actors in the dyad did not give a valid response to a question, it was treated as a missing value, which was the case for 20 (5 non-interviewed actors × 4) out of 1,056 ties for both networks, corresponding to a missing rate of <2%. If multiple respondents were interviewed from one unit, we used the responses given by the person highest in the hierarchy (11). Data were then managed and analyzed using UCINET 6. For data analysis, various descriptive and statistical procedures were applied. To identify actors' positions and key stakeholders, various centrality parameters (degree, betweenness, closeness, eigenvector) at the node level of analysis were calculated and assessed for all actors. For an analysis of structural cohesion at the network level, various measures of network cohesion were calculated (15, 41, 53): average degree (average number of edges per node in the graph), centralization (extent to which the graph shows a centralized structure), density (number of existing ties divided by the number of possible ties), fragmentation (extent to which the network is broken into fragments of unconnected nodes, dyads, and cliques), average distance [average number of steps along the shortest paths (geodesics) for all possible pairs of network nodes], and diameter (largest geodesic distance in the network). To analyze the association between the network of communication and the network of cooperation, inter-network correlations were calculated using the quadratic assignment procedure (QAP) (54). Network maps representing cooperation and communication between actors were visualized using GEPHI 0.9.2.

## Results

Respondents (*N* = 35) were asked to select from 13 different topics related to students' health that play a role in the course of their everyday professional lives. On average, each respondent selected six topics. Stress management (71% of all respondents), workplace design (63%), and key qualification and further education (63%) were mentioned most frequently, followed by sports and relaxation (60%), study organization (54%), social counseling (51%), study counseling (51%), curriculum (49%), campus design (46%), campus safety (40%), nutrition (29%), addiction counseling (17%), and health diagnostics (14%).

The network actors interpreted the question openly, which means that they assumed to have points of contact with the topics, even if they could not present any concrete offers themselves, but only referred students to offers of other actors. The respondents also found the response to the topics suitable if they were only relevant for a certain small part of the student body with whom they were in contact. Health-related topics mentioned additionally, once each, were health assessment, student representation possibility, sustainability, sleep, and peer-to-peer counseling. When asked to choose the topic, which plays the most important role in the everyday professional lives of the actors, respondents mentioned study organization (*n* = 4), sports and relaxation (*n* = 4), key qualification and further education (*n* = 3), workplace design (*n* = 3), study counseling (*n* = 3), and named once in each case: campus design, nutrition, health diagnostics, social counseling, campus safety, and sustainability. Eleven respondents did not make a statement in this regard, because they could not decide on 1 of the 11 topics.

When asked for the importance of the topics concerning students' health, respondents regarded stress management (M = 4.46, SD = 0.7), social counseling (M = 4.34, SD = 0.8), and sports and relaxation (4.23, SD = 0.9) as the most important topics, followed by workplace design (M = 4.11, SD = 0.9), study counseling (M = 4.00, SD = 1.1), study organization (M = 3.80, SD = 1.3), nutrition (M = 3.77, SD = 1.0), curriculum (M = 3.71, SD = 1.2), key qualification and further education (M = 3.69, SD = 1.1), addiction counseling (M = 3.57, SD = 1.0), campus design (M = 3.40, SD = 1.1), campus safety (M = 3.34, SD = 1.0), and health diagnostics (M = 3.20, SD = 1.0).

To assess how respondents view other actors in the network concerning students' health, respondents were asked to rate the importance of each actor. Respondents regarded the University Sports Center (M = 4.66, SD =.0.5), the Representative for Students with Special Needs (M = 4.51, SD = 0.6), and the Student Support Service (M = 4.46, SD = 0.9) as the most important actors (see Table 1). The mean ratings ranged between 2.24 and 4.66. Interestingly, some of the actors (e.g., Representative for Students with Special Needs, Study Center for Visually Impaired Students and Medical Services) deemed important here play a minor role in previous efforts to promote student health within the participatory health promotion project. This result corresponds to the network maps and structure constructs presented later.

**Table 1:** Importance of the units.

**Units**	**Mean (SD)**	***N***
University Sports Center	4.66 (0.5)	35
Representative for Students with Special Needs	4.51 (0.6)	35
Student Support Service	4.46 (0.9)	35
Corporate Health Management	4.35 (0.9)	34
Institute for Sports and Sports Science	4.29 (0.8)	35
Study Center for Visually Impaired Students	4.09 (0.9)	35
Presidium	4.03 (1.1)	35
Central Scientific Institution for Key Competencies	4.00 (0.7)	35
Sports Club	3.94 (0.9)	34
Medical Services	3.91 (1.1)	35
Student Group: Nightline	3.89 (1.1)	35
General Student Committee	3.66 (0.9)	35
Library and Learning Space Development	3.60 (1.2)	35
Equal Opportunities	3.59 (1.0)	34
Institutes	3.57 (1.0)	35
Service Unit for Higher Education and Student Affairs	3.52 (1.2)	33
Safety and Environment	3.52 (1.1)	33
Specialists for Occupational Safety	3.51 (1.1)	35
Student Groups	3.50 (1.0)	32
Center for Information and Counseling	3.44 (1.1)	34
Student Services	3.43 (1.1)	35
Student Parliament	3.37 (1.2)	35
Diversity Management	3.35 (1.1)	34
Campus Development	3.33 (1.1)	33
Student Working Group Culture and Communication	3.26 (1.1)	35
Deans' Offices	3.26 (1.2)	35
International Students Office	3.24 (1.2)	34
Student council Conference	3.06 (1.2)	35
Center for Applied Cultural Studies	2.91 (1.0)	35
Green-Alternative Student Group	2.86 (1.0)	35
Center for Teacher Education	2.79 (1.1)	33
Human Resources Development and Vocational Training	2.77 (1.2)	35
Innovation and Relations Management	2.24 (1.1)	33

Respondents were also asked to indicate the most important actor regarding the 11 health-related topics. The mentioned actors with the respective percentage number can be seen in Table 2 for every single topic. It can be seen that the perceived competence in terms of professional suitability and responsibility for a topic is distributed among different actors for each topic.

**Table 2:** Most competent units regarding the health-related topics.

**Topics**	**Most competent units**	***N***
Campus design	Campus Development (26%), Safety and Environment (20%), Facility Management (9%)	35
Curriculum	Institutes (38%), Deans' Office (24%), Service Unit for Higher Education and Student Affairs (18%)	34
Nutrition	Student Support Service (39%), Institute for Sports and Sports Science (27%), Corporate Health Management (15%)	33
Workplace design	Specialists for Occupational Safety (27%), Library and Learning Space Development (21%), Facility Management (12%)	34
Health diagnostics	Institute for Sports and Sports Science (88%), University Sports Center (6%)	33
Key qualification and further education	Central Scientific Institution for Key Competencies (65%), Human Resources Development and Vocational Training (12%)	34
Social counseling	Student Support Service (43%), General Student Committee (17%), Study Center for Visually Impaired Students (9%)	35
Sports and relaxation	University Sports Center (54%), Institute for Sports and Sports Science (37%)	35
Stress management	Student Support Service (25%), Central Scientific Institution for Key Competencies (25%), Institute for Sports and Sports Science (16%), Corporate Health Management (16%)	32
Study Counseling	Center for Information and Counseling (65%), Student Services (12%)	34
Study Organization	Service Unit for Higher Education and Student Affairs (36%), Institutes (18%), Presidium (12%), Student Services (12%)	33
Addiction Counseling	Student Support Service (59%), Medical Services (25%)	32
Campus safety	Presidium (36%), Safety and Environment (30%)	33

*Due to lack of space, single mentions have not been displayed*.

Furthermore, respondents were asked if there were any actors not included in this survey that they considered to play a significant role regarding students' health. Fourteen of the 35 respondents (40%) named at least one additional actor. The nominations are as follows: Facility Management (number of mentions: 6), General Services (4), Faculties (3), Conflict Management and Psychosocial Counseling (2), Student Councils (2), Service Unit for University Law and Academic Affairs (1), University Departments (1), Service Unit for Law (1), Adjunct Lecturers (1), Strategic Corporate Development and Communications (1), Canteen (1), Study Commission (1), Faculty Council (1), Physics Student Council (1), Social Club in the Student House (1), Center for Technology-Enhanced Learning (1), Representative for Refugees (1), and Vice-President for Higher Education and Student Affairs (1). Thus, 18 actors that were previously less in the focus of the participatory health promotion project but could play a meaningful role in improving students' health have been identified. Facility Management, General Services, and Faculties were mentioned by multiple respondents and are thus ideal targets for engagement efforts in the future.

**Figure 1:**
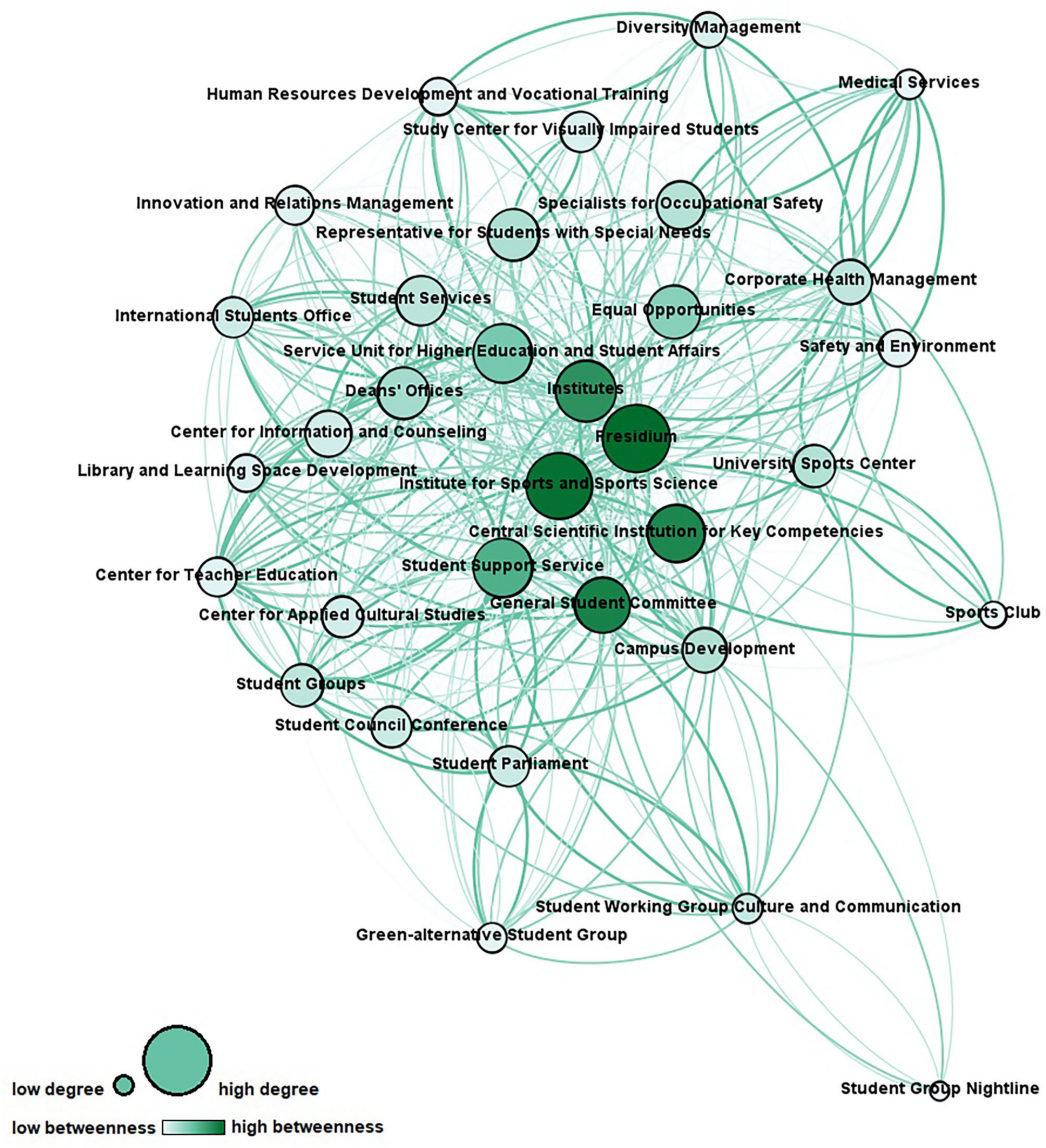
Cooperation network (node size represents degree centrality; node color represents betweenness centrality; link thickness and color represent intensity of cooperation). Network measures for the cooperation network are reported in Table 3.

**Figure 2:**
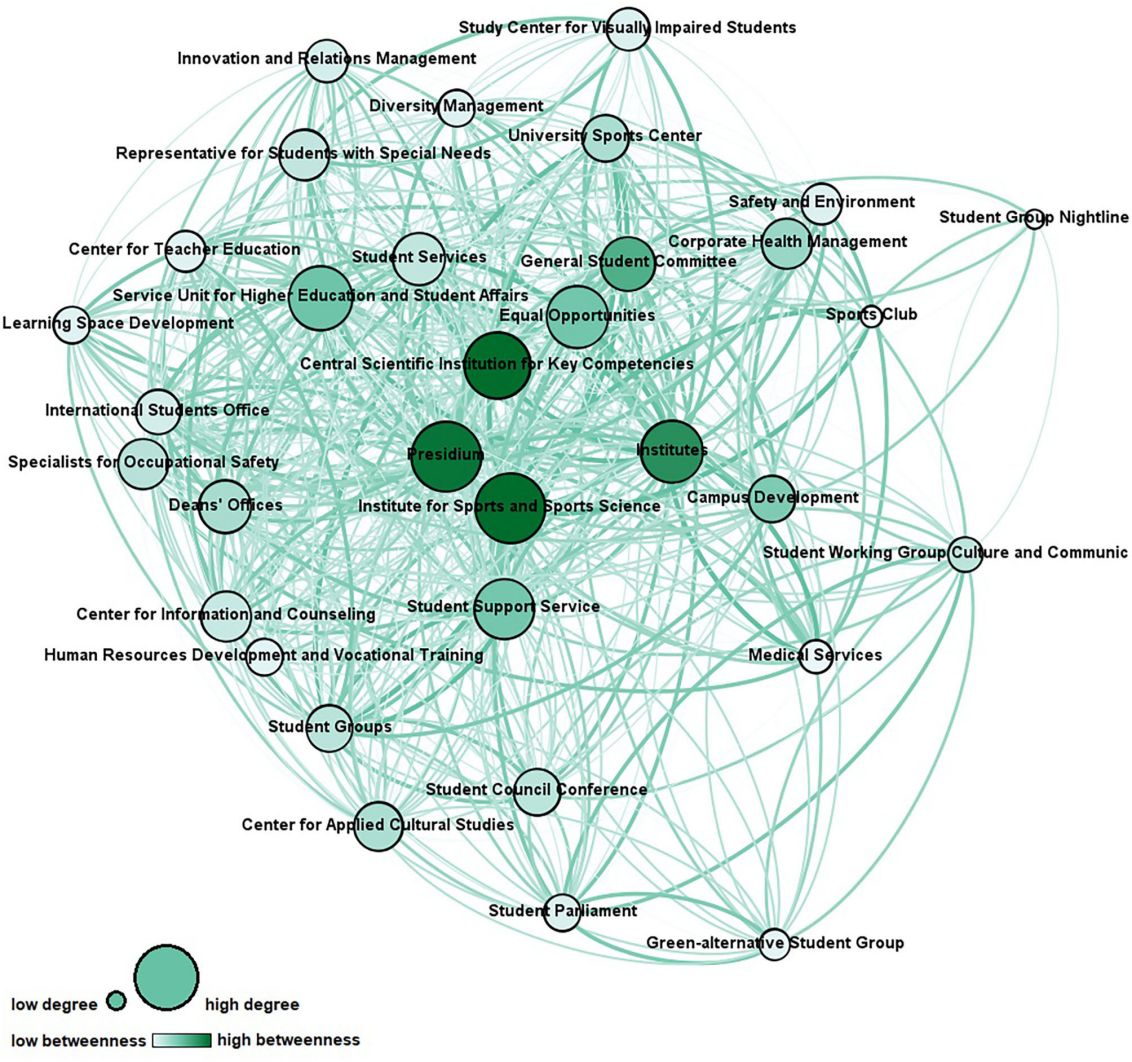
Communication network (node size represents degree centrality; node color represents betweenness centrality; link thickness and color represent frequency of contact). Network measures for the communication network are reported in Table 4.

Respondents were asked to rate their level of cooperation and communication with each actor from the list. Two network maps were generated from these variables for analysis. The first network map shows the cooperation linkages (Figure 1), and the second network map shows the communication linkages (Figure 2). Reciprocity of the original dataset was ~0.5. Using the QAP procedure, there is a significant positive high correlation with *r* = .85 (*p* < 0.05) between the cooperation network with the communication network.

**Table 3:** Network Measures of the cooperation network (dichotomized data).

**Measures**	**Cooperation network**
Number of nodes	33
Number of ties	560
Average degree	16.97
Degree centralization	0.433
Density	0.53
Fragmentation	0
Average distance	1.473
Standard deviation Distance	0.507
Diameter	3

**Table 4:** Network Measures of the communication network (dichotomized data).

**Measures**	**Communication network**
Number of nodes	33
Number of ties	600
Average degree	18.182
Degree centralization	0.393
Density	0.568
Fragmentation	0
Average distance	1.434
Standard deviation distance	0.499
Diameter	3

In terms of the cooperation network, 560 out of 1,056 possible ties of the network were realized, resulting in a density of 0.53. Almost half of these ties (228, or 41%) suggested a cooperation level of information sharing only, while the other cooperation levels were as follows: informal cooperation (92, or 16%), formal cooperation (160, or 29%), and partnership (80, or 14%).

In terms of the communication network, 600 out of 1,056 possible ties of the network were realized, resulting in a density of 0.57. Ninety-two of these ties (15%) suggested a communication level of less than annually, while the other communication levels were as follows: annually (98, or 16%), half-yearly (202, or 34%), monthly (108, or 18%), weekly (74, or 12%), daily (16, or 3%).

To identify key stakeholders in the original cooperation and communication networks, the following network structure constructs on actor level have been calculated (55–57):

- Degree centrality: to explore who is a central connector by means of the number of ties an actor has with others and can be considered prestigious and influential;
- Betweenness centrality: to explore who is a gatekeeper or information broker and connects various nodes in the network and therefore supports information exchange and has control over the network communication;
- Closeness centrality: to explore who is an autonomous actor and therefore close to all other actors based on the distance between nodes so that he can spread information efficiently; and
- Eigenvector centrality: to explore who is a popular actor by means of the number of ties an actor has with other high-scoring actors concerning centrality.

An overview of the scores for the most central actors can be found in Table 5.

**Table 5:** Overview of the network measure scores for the individual actors in the cooperation and communication network.

**Cooperation network**	
Most influential actors based on degree	1. Presidium (85), 2. Institute of Sports and Sports Science (71), 3. Institutes (65)
Information brokers based on betweenness	1. Presidium (28.7), 2. Institute of Sports and Sports Science (27.9), 3. General Student Committee (25.0)
Most integrated actors based on closeness	1. Presidium (34), 2. Institute of Sports and Sports Science (35), 3. Institutes (38)
Most popular actors based on eigenvector	1. Presidium (1), 2. Institute of Sports and Sports Science (0.86), 3. Institutes (0.79)
**Communication network**	
Most influential actors based on degree	1. Presidium (114), 2. Institute of Sports and Sports Science (100), 3. Institutes (98)
Information brokers based on betweenness	1. Central Key Qualification Facility (25.4), 2. Institute of Sports and Sports Science (25.3), 3. Presidium (24.2)
Most integrated actors based on closeness	1. Presidium (34) and Institute of Sports and Sports Science (34), 3. Central Scientific Institution for Key Competencies (36)
Most popular actors based on eigenvector	1. Presidium (1), 2. Institute of Sports and Sports Science (0.89), 3. Institutes (0.87)

To explore who is a *decentral specialist* providing specific knowledge, but is peripheral in the network, a comparison of the actors' legitimacy and competency attributions regarding students' health (see Tables 1, 2) with their centrality scores has been made. Medical Services, the Student Group Nightline, the Sports Club, the Specialists for Occupational Safety, and the Center for Information and Counseling were identified as such.

## Discussion

### Summary of Main Findings

The network analysis at hand provides in-depth insights into university structures promoting students' health comprising 33 organizational units and hundreds of ties. Both cooperation and communication network show a flat, non-hierarchical structure, which is typical for the university context (58). This structure is reflected by its low centralization indices and short average distances with low standard deviations, indicating that every actor can be reached by every other actor *via* one to two nodes as a rule. The largest geodesic distance in the network, which is expressed by diameter, is small, and with regard to fragmentation, the networks show the non-existence of subgroups. Density, in other words the ratio of observed ties to the number of possible ties, is relatively high. It is assumed that high density increases the probability that weak ties turn into strong ties in the future (59). Every node is connected with more than half of the networks' nodes on average, which is expressed by average degrees. Due to the compactness and connectedness of the network, it can be assumed that information is likely to reach everyone in the network quickly. The pattern of linkages of the cooperation network suggests that the highest number of relations among the actors were for information sharing. This finding is consistent with previous research on public health networks, which shows that stakeholders tend to communicate rather than cooperate as this is associated with less effort (60). The cooperation network and the communication network are highly correlated (*r* = .85, *p* < 0.05), showing that these two networks are not independent of each other. Simultaneously the density of the cooperation network is less pronounced than the density of the communication network. This is in line with current research findings, which show that communication can be considered a precursor to cooperation (54, 61). From network analyses in other settings, it is furthermore known that actors tend to form ties with similar ones because of the similar nature of work (16, 49). This phenomenon is called homophily (62) and can partly be observed within the present network (e.g., interconnectedness of the student groups).

### Interpretation of Findings

Substantial cooperation between university actors with very different core agendas is needed for health promotion of university students (4, 33). Since it is a young field of activity with an unclear role distribution, university units may have limited experience at cooperating in this regard. The present findings allow identifying starting points for effective network development and governance in revealing key stakeholders as well as in discovering actors that should take on a significant role in the future process. Across the two networks, opportunities for further integration and interaction exist. According to the respondents, the University Sports Center, the Representative for Students with Special Needs, and the Corporate Health Management are among the most important actors regarding students' health. However, they only play a minor role in the cooperation and communication network thus far. Interestingly, four of the top 10 actors (see Table 1) have chosen sports and relaxation as the topic, which plays the most important role in their everyday professional lives, suggesting that this classic field of action of health promotion is of key importance in regard to promoting students' health. Still, the network actors cover all requested health-related topics, and it is noteworthy that topics that constitute the core business of universities (e.g., key qualification and further education, study counseling and curriculum) are not considered unimportant in the context of health promotion for students, which opens the possibility to integrate the topic of health crosswise at the university. Concerning cross-linkage of actors who contribute to the same health-related topic, strong relationships should be established, so that the division of tasks can be clearly defined and synergies created. Except for the General Student Committee, student groups tend to be located on the periphery of the network with fewer ties than central actors. Looking to the future, it will be important to find out under what circumstances it is desirable and achievable for them to be more integrated in order to ensure that they participate in the health promotion process and that their needs and requirements are adequately addressed. Besides, opportunities to strengthen the ties of *decentral specialists* are evident. The integration of distal nodes may lead to new insights and offers new input for the matter (63). Medical services, in particular, could take on a much more significant role with regard to student health in the future as part of the risk assessment of mental stress. Stakeholders from the participatory health promotion project for students (e.g., Presidium, Institute of Sport and Sports Science, or Central Scientific Institution for Key Competencies) play an integral role in both networks. The data confirm that the project already operates with key stakeholders and suggest to continue engaging these actors in activities for health promotion. Presidium and Institute of Sport and Sports Science are the most important actors in terms of the functionality in the network (see Table 5). The commitment of the presidium of a university, in particular, is regarded as a crucial factor for the success of health promotion efforts regarding students, and health-related disciplines can provide important impetus in the process (40, 64). Institutes should be involved in health promotion efforts in their position as multipliers with direct contact to all students. Besides, barriers to cooperation, for example, bureaucracy, differing goals or agendas of units, lack of time, and previous experiences of working together, should be considered in the development of the health promotion network (16, 49). For example, formal agreements could be used to determine goals in advance and define responsibilities for cooperation in this way to prevent the fear of a loss of autonomy and an impoverishment of resources on the part of the individual actors.

Theoretical papers in the context of health-promoting universities recommend the creation of an organizational structure to coordinate all actions related to health (40). While this is probably the first network that was analyzed this profoundly in the university setting on behalf of students' health, research from other fields allows concluding effective modes of network development and governance that can be applied in the context of a university. Goal-directed networks, such as the actor network of health-promoting universities, require a certain form of governance to utilize the benefits of cooperation among stakeholders (65). The network at hand shows characteristics of a “participant-governed” network, which is governed by virtually all involved units coordinating activities and making decisions (although stakeholders of the participatory health promotion project play a special role in it as a kind of “leading group”). Such networks are common in the field of health services to build community capacity (66). However, thought could still be given to whether a change in the governance approach might be useful. In “lead organization-governed” networks, for example, the network is led and coordinated by a legitimized central actor trusted by others (65). This form of governance also works with low commitment levels of the network members and is best suited for a moderate number of involved actors. To increase the efficiency of the network, a “network administrative organization” can also be considered, where governance is carried out externally by an independent unit, which is specifically set up to govern the network only (65). This approach best fits networks with moderate density and centralization, moderate to many network participants, and a moderately high goal consensus.

### Limitations and Transferability

The survey questions and response items may have limitations. For example, it may be challenging to rate the level of cooperation or communication with another organization on the whole. The reputational snowball sampling could have biased the boundary specification, and therefore the sample. Having two different key informants might have led to a different list of actors. In terms of validity, the survey included a question regarding additional actors, and the evaluation on this matter did not suggest that significant units were missing from the network sample, except for the Facility Management and General Services. Usual concerns about the use of informants, who may have only partial knowledge about the underlying issue, were not a concern in this study, since, in general, the units' executive director or, in some cases, a more knowledgeable staff member has been interviewed. Anyway, a bias in reporting or from missing data is a possible limitation in network analysis with key informant interviews (11). In particular, the consistent consideration of multiple actors from each unit could have had an impact on the results of the network analysis. Apart from that, certain actors could have been ruled out through a selection bias since isolated actors have no network at all (67). Reciprocity of the original dataset was ~0.5, reflecting uncertainty among respondents regarding the actual occurrence and magnitude of the relationships. The network analysis at hand included unconfirmed links, because using confirmed links only may underestimate the extent of cooperation (68). Minimization as an often-used symmetrizing approach was used to resolve rating discordances between two actors in a dyad conservatively (53). This first-time network analysis of health-promoting structures regarding students' health at a university maps hundreds of actor ties and reflects the views of dozen units, but since the analysis is limited to the health promotion network at one single university, generalizations based on the available data should be made with caution. However, the fact that administrative structures of universities are basically comparable, at least in Germany and in the European higher education area (69, 70), allows for a transfer of the numerous indications for network development, such as:

- University executive board and health-related disciplines as key stakeholders;
- Crosswise integration of health promotion *via core-business*-units of university;
- Utilizing the potential of subordinate stakeholders (e.g. *decentral specialists*);
- Informed decision on network governance of the health-promoting network;
- Representation of student groups' participation *via* cooperation in the network; and
- Academic stress as focal point within health promotion for university students.

### Future Direction and Conclusion

The present work has laid a foundation for future research that could include a longitudinal evaluation of the network by collecting data once again with the inclusion of the additional actors identified by respondents. Thereby, assessment should be extended by meaningful constructs (e.g., funding flow or resource sharing) to gain deeper insight into the network and by structural contingencies (e.g., network goal consensus or trust) to predict the effectiveness of network governance. Network analysis can thereby represent a new form of structure evaluation in health promotion, in which the emphasis is less on simple counts of program activities and more on the documentation of structural changes (11). Compared to other methods of identifying key stakeholders, network analysis is characterized by high validity and reliability as well as being time-consuming and resource-intensive (71). On a final note, this form of data collection enables universities to profoundly analyze their health-promoting structures, which is the basis for sustained network governance and development.

## Data Availability Statement

The datasets generated for this study are available on request to the corresponding author.

## Ethics Statement

Ethical review and approval was not required for the study on human participants in accordance with the local legislation and institutional requirements. The patients/participants provided their written informed consent to participate in this study.

## Author Contributions

All authors listed have made a substantial, direct and intellectual contribution to the work, and approved it for publication.

## Conflict of Interest

The authors declare that the research was conducted in the absence of any commercial or financial relationships that could be construed as a potential conflict of interest.
